# Short-term antithrombotic strategies after left atrial appendage occlusion: a systematic review and network meta-analysis

**DOI:** 10.3389/fphar.2023.1159857

**Published:** 2023-09-01

**Authors:** Li-Man Wang, Yan Chen, Li-Li Xu, Meng-Fei Dai, Yi-Jun Ke, Bao-Yan Wang, Lin Zhou, Ji-Fan Zhang, Zhang-Qi Wu, Yu-Jie Zhou, Zhi-Chun Gu, Hang Xu

**Affiliations:** ^1^ Department of Pharmacy, China Pharmaceutical University Nanjing Drum Tower Hospital, Nanjing, China; ^2^ School of Basic Medicine and Clinical Pharmacy, China Pharmaceutical University, Nanjing, China; ^3^ Department of Pharmacy, Anqing Municipal Hospital, Affiliated with Anhui Medical University, Anqing, China; ^4^ Nanjing Foreign Language School, Nanjing, China; ^5^ Nanjing Jinling High School International Department, Nanjing, China; ^6^ Department of Respiratory and Critical Care Medicine, Nanjing Drum Tower Hospital, The Affiliated Hospital of Nanjing University Medical School, Nanjing, China; ^7^ Department of Pharmacy, Ren Ji Hospital, Shanghai Jiao Tong University School of Medicine, Shanghai, China

**Keywords:** left atrial appendage occlusion, network meta-analysis, warfarin, dual antiplatelet therapy, direct oral anticoagulants

## Abstract

**Background:** Percutaneous left atrial appendage occlusion (LAAO) has emerged as a stroke prevention strategy in patients with nonvalvular atrial fibrillation (NVAF), and these patients were required to receive antithrombotic therapy post-procedure. However, the optimal antithrombotic strategy after LAAO remains controversial. This study explored the safety and efficacy of different antithrombotic strategies after LAAO through a network comparison method.

**Methods:** We systematically searched the MEDLINE, Embase, and Cochrane Library databases for studies that reported the interested efficacy and safety outcomes (stroke, device-related thrombus (DRT), and major bleeding) of different antithrombotic strategies [DAPT (dual antiplatelet therapy), DOACs (direct oral anticoagulants), and VKA (vitamin k antagonist)] in patients who had experienced LAAO. Pairwise comparisons and network meta-analysis were performed for the interested outcomes. Risk ratios (RRs) with their confidence intervals (CIs) were calculated using a random-effects model. The rank of the different strategies was calculated using the surface under the cumulative ranking curve (SUCRA).

**Results:** Finally, 10 observational studies involving 1,674 patients were included. There was no significant difference in stroke, DRT, and major bleeding among the different antithrombotic strategies (DAPT, DOACs, and VKA). Furthermore, DAPT ranked the worst in terms of stroke (SUCRA: 19.8%), DRT (SUCRA: 3.6%), and major bleeding (SUCRA: 6.6%). VKA appeared to be superior to DOACs in terms of stroke (SUCRA: 74.9% vs. 55.3%) and DRT (SUCRA: 82.3% vs. 64.1%) while being slightly inferior to DOACs in terms of major bleeding (SUCRA: 71.0% vs. 72.4%).

**Conclusion:** No significant difference was found among patients receiving DAPT, DOACs, and VKA in terms of stroke, DRT, and major bleeding events after LAAO. The SUCRA indicated that DAPT was ranked the worst among all antithrombotic strategies due to the higher risk of stroke, DRT, and major bleeding events, while VKAs were ranked the preferred antithrombotic strategy. However, DOACs are worthy of consideration due to their advantage of convenience.

## 1 Introduction

Nonvalvular atrial fibrillation (NVAF) is the most common arrhythmia in the middle-aged and elderly and is associated with an increased risk of stroke and thromboembolic events. Studies showed that more than 90% of thromboembolic events originate from the left atrial appendage in patients with NVAF because of the influence of anatomical location and function (Gallinoro et al., 2019; Holmes et al., 2019). Percutaneous left atrial appendage occlusion (LAAO) has gradually emerged as an effective treatment strategy for patients with NVAF (Cimmino et al., 2021). However, despite the surgeon’s experience and device technology having significant improvements, LAAO is still associated with the risks of potentially serious stroke, device-related thrombus (DRT), and major bleeding, which was the same as most implantation procedures (Yu et al., 2021). This is because when a foreign material is placed into the human system during the LAAO procedure, thrombosis may occur on the device surface contributing to thromboembolic events before adequate endothelialization (Price, 2019). Therefore, antithrombotic therapy is essential for patients undergoing LAAO (Mahajan et al., 2012). Although the U.S. Food and Drug Administration labeling for the WATCHMAN device recommended using 45 days of warfarin followed by 6 months of DAPT (Jalal et al., 2017; Saw et al., 2019), practitioners rarely used the approved treatment protocols when using the WATCHMAN device in clinical practice, while some prefer dual antiplatelet therapy or direct oral anticoagulants (Reddy et al., 2017; Boersma et al., 2019). In addition, DOACs are favored for stroke prevention because of better safety and convenience compared with warfarin (Connolly et al., 2009; Giugliano et al., 2013; Carnicelli et al., 2022), whereas some studies show that there seem to be similar risks of thromboembolism and bleeding between different antithrombotic strategies in patients after LAAO (Boersma et al., 2019; Osman et al., 2020). Currently, there are no published systematic reviews on randomized controlled trials or observational studies comparing all commonly used antithrombotic strategies. The optimal antithrombotic strategies for patients undergoing LAAO remain controversial and, hence, require further exploration. Therefore, we decided to conduct a network meta-analysis to systematically explore the safety and efficacy of different antithrombotic strategies after LAAO to provide credible evidence for clinical decision-making.

## 2 Methods

### 2.1 Search strategy

The study was conducted according to the standards of the Preferred Reporting Items for Systematic Reviews and Meta-Analyses (PRISMA) statement PROSPERO registry with the registration number CRD42022304389 (Moher et al., 2015). The Cochrane Library, MEDLINE, and Embase databases were systematically searched for studies that directly compared different antithrombotic strategies (DAPT, DOACs, and VKA) after LAAO. All the English publications until October 2022 were searched. For the theme “Left Atrial Appendage Occlusion,” the terms used were “Left Atrial Appendage Occlusion” OR “Left atrial appendage closure.” For the theme “Platelet Aggregation Inhibitors,” we included the following terms: “Aspirin” OR “Ticlopidine” OR “Clopidogrel” OR “Dipyridamole” OR “Thienopyridines.” For the theme “Anticoagulants,” we included the following terms: “Warfarin” OR “Non-vitamin K antagonist oral anticoagulants” OR “NOACs” OR “Direct oral anticoagulants” OR “DOACs” OR “Novel oral anticoagulants” OR “New oral anticoagulants” OR “Factor Xa inhibitors” OR “Rivaroxaban” OR “Xarelto” OR “Edoxaban” OR “Lixiana” OR “Savaysa” OR “Apixaban” OR “Eliquis” OR “Dabigatran” OR “Pradaxa.” We used the Boolean operator “AND” to combine the three comprehensive search themes. To confirm articles that were missed in the early search, the reference list of each paper was filtered. In addition, unpublished data were obtained from the ClinicalTrials.gov website.

### 2.2 Inclusion criteria and exclusion criteria

The following were the inclusion criteria: 1) observational studies; 2) studies that enrolled patients who received LAAO device implantation (WATCHMAN, Amulet, or Amulet Cardiac Plug [ACP]); 3) studies that adopted specific antithrombotic regimens after LAAO; and 4) studies that explicitly reported the detailed information about the safety and efficacy outcomes of patients. The following studies were excluded: 1) studies with fewer than 10 subjects; 2) studies without follow-ups; 3) studies with duplicate or lost data; and 4) case reports, reviews, conference abstracts, and guidelines. We also excluded the subsequent studies or sub-studies based on similar study cohorts. In addition, for multiple publications based on the same patient pool, we only included the most recent published articles.

### 2.3 Study outcomes

The primary efficacy and safety outcomes were stroke, device-related thrombus (DRT), and major bleeding. The stroke events were defined as all-cause strokes (ischemic or hemorrhagic) following implantation. The DRT events were defined as thrombosis on the atrial surface of the device visible through transesophageal echocardiography (TEE) or CT scan (Korsholm et al., 2019). Furthermore, the major bleeding events included a decrease in the hemoglobin level of 2 g/dL or greater within a 24-h period or leading to a transfusion of 2 or more units of packed red cells or requiring an additional endoscopy intervention, according to the International Society on Thrombosis and Hemostasis (ISTH) criteria (Mega et al., 2009).

### 2.4 Data extraction

We used a pre-customized form to extract and collect data from the included studies. The data extracted from each study included characteristics of the individual study (study name, year of publication, number of patients, antithrombotic strategy, duration of follow-up, and study design), the baseline characteristics of patients (age, sex, type of atrial fibrillation, heart failure, hypertension, diabetes mellitus, CHA_2_DS_2_-VASc score, and HAS-BLED score), and the information of the interested outcomes (stroke, DRT, and major bleeding).

### 2.5 Quality assessment

Quality assessment of the enrolled observational studies was performed via the Newcastle–Ottawa Scale (NOS) (GA Wells, 2021). This scale was divided into NOS evaluation criteria for cohort studies and for case–control studies. The scale consists of three major parts (evaluation of selection, comparability, and outcome), using a star system, with full marks of 13 stars for cohort studies and nine stars for case–control studies. We conducted this quality assessment using the evaluation criteria for cohort studies. Studies with at least six stars were included in the meta-analysis.

### 2.6 Statistical analysis

The extraction form of effects was events for dichotomous data and means or median for continuous data. These data were recorded directly according to the study data or computed according to the data provided in the study. To estimate the pooled relative risk (RR) with 95% confidence intervals (CIs), we first performed a pairwise meta-analysis using Stata 15.1. A value of *I* ^2^ ≥50% was considered substantial heterogeneity. When there was statistically significant heterogeneity, a random-effects model was used; otherwise, a fixed-effects model was used. In addition, we performed network meta-analysis and assessment of inconsistency using the command “mvmeta” of the Stata Statistical Software 15.1. Inconsistency of the indirect and direct evidence was assessed using the heterogeneity variance parameter (tau-squared, τ^2^) in the loop-specific approach, which assesses the bias of effect sizes among the study participants. At least three treatment pairs are required to form an evidence loop. Probability values were shown as the surface under the cumulative ranking (SUCRA) curve and provided a rank of antithrombotic strategies; the SUCRA value becomes 0% when it is certain to be the worst and 100% when it would be the best. The robustness of treatment effects in different antithrombotic strategies was evaluated by meta-regression in direct comparative treatment subgroups using the proportion of the device type. Moreover, we used comparison-adjusted funnel plots to observe the potential publication bias among the studies that were included. *p*-values of less than 0.05 were considered statistically significant.

## 3 Results

### 3.1 Study selection

**FIGURE 1:**
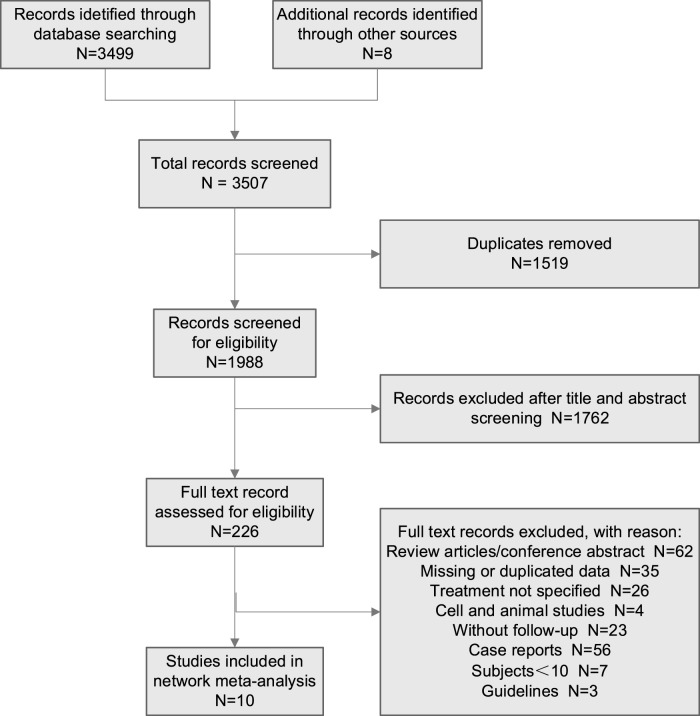
Flow diagram for the selection of eligible studies.

A total of 3,507 studies were initially retrieved. After excluding duplicate studies, 1,988 studies were screened for eligibility for further scanning. Then, a total of 226 studies were assessed for eligibility using the preordained selection criteria. Through reading the abstract and browsing the partial text of the articles, 216 studies were excluded according to the exclusion criteria. Finally, 10 studies that met the inclusion criteria were enrolled in this network meta-analysis **(** Figure 1 **)**.

### 3.2 Quality assessment

All 10 comparative studies were assessed for quality, with NOS scores ranging from 6 to 9, which indicated that the studies included were moderate to high-quality studies. The quality assessment results of all included studies are shown in Supplementary Table S1.

### 3.3 Characteristics of the studies

A total of 10 studies with 1,674 patients were enrolled in this meta-analysis (Bösche et al., 2015; Wiebe et al., 2015; Kim et al., 2016; Enomoto et al., 2017; Cohen et al., 2019; Duthoit et al., 2019; Cepas-Guillen et al., 2021; Chen et al., 2021; Faroux et al., 2021; Zhu and Xu, 2021). The patients were divided into three groups according to the antithrombotic strategies they received: DAPT (aspirin + clopidogrel/ticlopidine), DOACs (rivaroxaban/dabigatran), and VKA (warfarin). The incidence of stroke and major bleeding were both reported in nine studies and DRT in six studies. Of the 10 studies, five compared DOACs and DAPT, four compared DOACs and VKA, and only one compared DAPT and VKA **(** Supplementary Figure S1 **)**. This analysis mostly included elderly patients with hypertension as the main complication. The detailed baseline characteristics are presented in Table 1 **.**

**TABLE 1:** Summarized characteristics of the included studies.

Study		Bösche 2015	Cepas-Guillen 2021	Duthoit 2019	Faroux 2021	Kim 2016	Wiebe 2015	Jing Zhu 2021	Enomoto 2017	Chen 2021	Cohen 2019
Age (mean ± SD)		75 ± 7	73.1 ± 9	77.5 ± 8.2	75.9 ± 8.1	65.1 ± 9.4	71.6 ± 8.8	66 (46–86)	75.5 ± 8	64.8 ± 8.2	76.9 ± 8.7
Male (%)		58.0%	65.0%	62.5%	58.2%	61.5%	62.7%	58.6%	34.0%	NR	62.9%
Hypertension		91.0%	91.0%	85.6%	90.5%	70.8%	91.2%	68.6%	NR	NR	89.7%
Diabetes mellitus		34.00%	NR	27.90%	33.00%	38.50%	27.50%	20.00%	NR	16.2%	21.60%
Previous stroke/TIA		31.0%	43.0%	48.1%	38.6%	43.8%	40.1%	74.2%	NR	42.6%	40.2%
Coronary artery disease		53%	NR	10.6%	NR	38.5%	NR	41.4%	NR	33.5%	NR
CHA_2_DS_2_-VASc score (mean ± SD)		4.0 ± 1.4	4.3 ± 1.5	4.5 ± 1.5	4.6 ± 1.6	3.9 ± 1.6	4.3 ± 1.7	3.9 ± 1.0	3.9 ± 1.6	3.1 ± 1.7	4.7 ± 1.5
HAS-BLED score (mean ± SD)		3.5 ± 0.8	3.6 ± 1.0	3.7 ± 1.0	3.6 ± 1.0	2.7 ± 1.3	2.9 ± 1.2	3.2 ± 0.8	2.5 ± 1.1	1.8 ± 1.2	3.5 ± 1.0
Follow-up (month)		1.5	3	3	3	22	6	1.5	4	1.5	8
Therapeutic regimen	DAPT	27	73	33	190	35	41				
	DOACs	18	40	71	95	61		30	212	170	52
	VKA						57	40	214	170	45

### 3.4 Pairwise comparison

The results of direct comparisons are shown in Supplementary Figure S2. Compared with DOACs, DAPT did not show an increased risk of stroke (RR 1.83; 95% CI, 0.44–7.63; *p* = 0.56), DRT (RR 4.07; 95% CI, 0.51–32.18; *p* = 0.94), and major bleeding (RR 1.54; 95% CI, 0.82–2.89; *p* = 0.45). Similarly, treatment with DAPT was not associated with a significantly increased risk of stroke, DRT, and major bleeding compared with VKA. Moreover, no statistically significant difference was found between DOACs and VKA regarding stroke, DRT, and major bleeding.

### 3.5 Network meta-analysis

**FIGURE 2:**
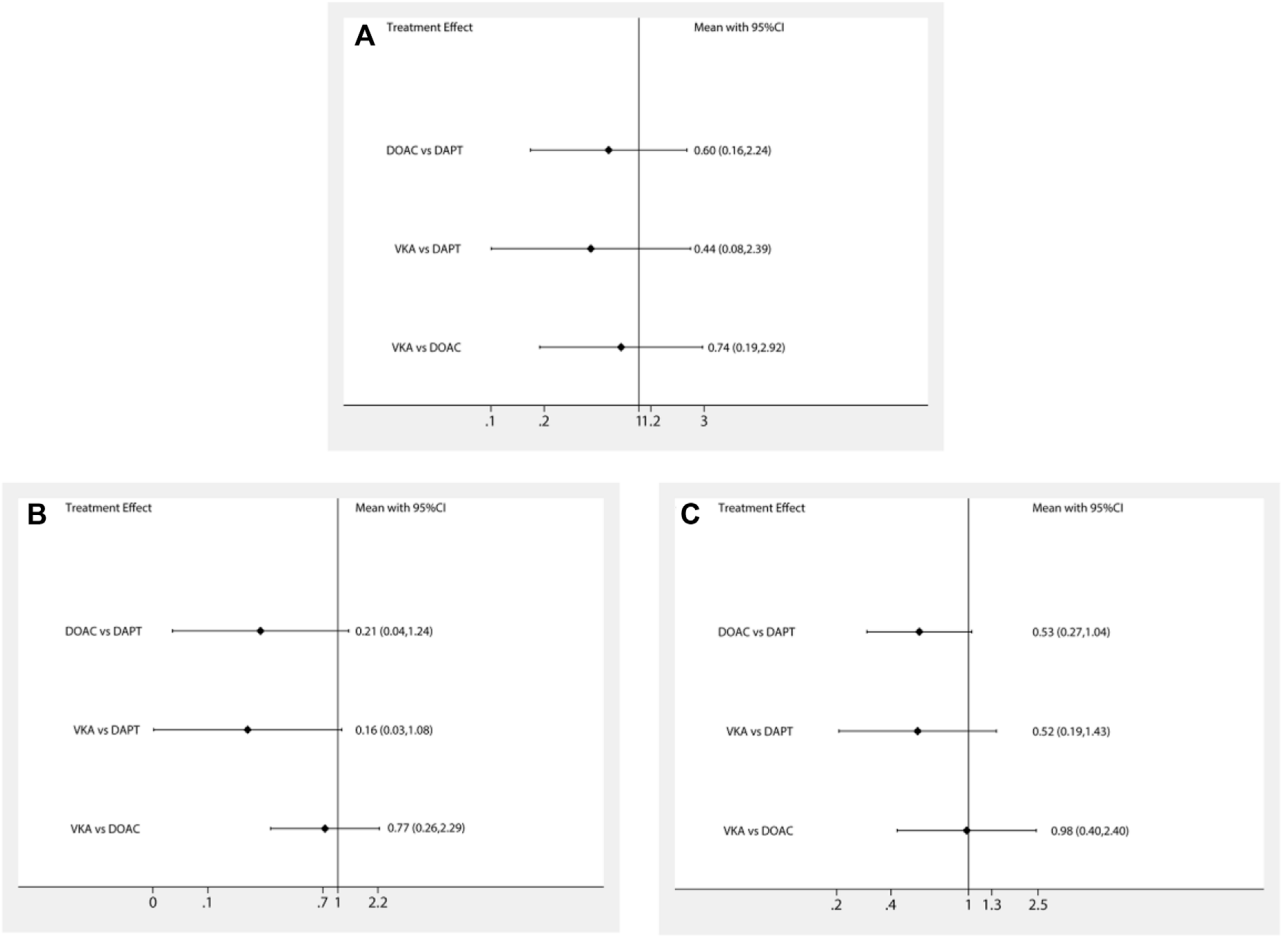
Forest plot for the network meta-analysis of all outcomes: **(A)** strokes, **(B)** DRT, and **(C)** major bleeding.

The network meta-analysis results are presented in Figure 2. In terms of stroke, there was no significant difference between patients treated with DAPT, DOACs, and VKA after LAAO (DOACs vs. DAPT: RR 0.60; 95% CI, 0.16–2.24; VKA vs. DAPT: RR 0.44; 95% CI, 0.08–2.39; VKA vs. DOACs: RR 0.74; 95% CI, 0.19–2.92). Furthermore, in terms of DRT and major bleeding, no significant difference was found among all strategies. Finally, there was no significant difference in stroke, DRT, and major bleeding among the different antithrombotic strategies (DAPT, DOACs, and VKA) after LAAO. Similar results were observed in the pairwise comparison (Supplementary Figure S2).

#### 3.5.1 Rank probability

The SUCRA and absolute rank probabilities of antithrombotic strategies are shown in Table 2. In terms of stroke, DAPT (SUCRA: 19.8%) had the lowest cumulative ranking probability and VKA (SUCRA: 74.9%) had the highest cumulative ranking probability, followed by DOACs (SUCRA: 55.3%). With respect to DRT, compared with VKA (SUCRA: 82.3%) and DOACs (SUCRA: 64.1%), DAPT (SUCRA: 3.6%) ranked the worst. In regards to major bleeding, DOACs (SUCRA: 72.4%) had the highest cumulative ranking probability, followed by VKA (SUCRA: 71.0%) and DAPT (SUCRA: 6.6%). VKA was the most effective treatment, and DOACs were the safest in patients who experienced LAAO. VKA had similar safety patterns to DOACs.

**TABLE 2:** Surface under the cumulative ranking of the primary outcome.

Intervention	Stroke			DRT			Major bleeding		
	SUCRA (%)	PrBest	MeanRank	SUCRA (%)	PrBest	MeanRank	SUCRA (%)	PrBest	MeanRank
DAPT	19.8	9.6	2.6	3.6	1.8	2.9	6.6	1.5	2.9
DOACs	55.3	28.0	1.9	64.1	31.7	1.7	72.4	46.7	1.6
VKA	74.9	62.4	1.5	82.3	66.6	1.4	71.0	51.8	1.6

DAPT, dual antiplatelet therapy; DOACs, direct oral anticoagulants; VKA, vitamin K antagonist; SUCRA, surface under the cumulative ranking.

#### 3.5.2 Trade-off analysis

**FIGURE 3:**
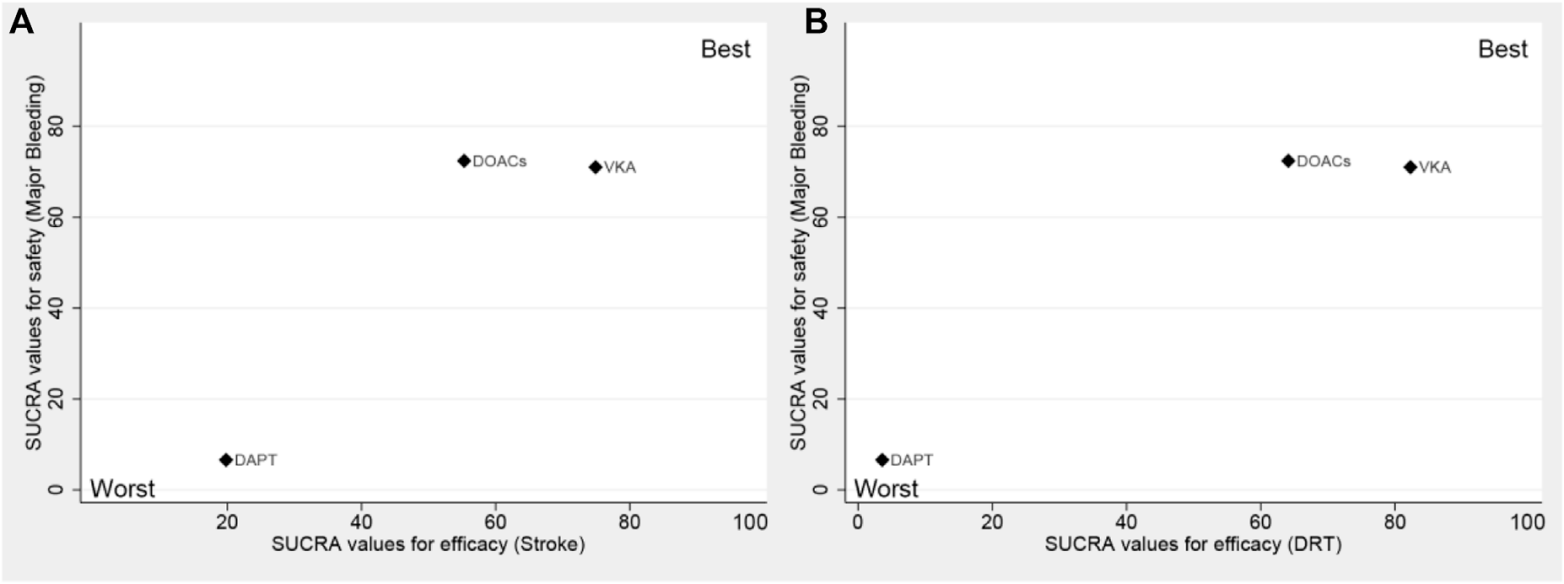
Surface under the cumulative ranking (SUCRA) plot. Ranking of strategies expresses the probability associated with each one being the best with respect to stroke and major bleeding **(A)**, as well as DRT and major bleeding **(B)**. The strategies in the upper right corner are more effective and safer than the other strategies. DAPT, dual antiplatelet therapy; DOACs, direct oral anticoagulants; VKA, vitamin K antagonist.

Trade-off analyses of different antithrombotic strategies are shown in Figure 3. The clustered ranking plot according to SUCRA values indicated that DAPT occupied the most unfavorable position with respect to efficacy and safety. VKA formed a cluster of “the most effective and reasonably safe” treatment, whereas DOACs presented a cluster of “the most safe and reasonably effective” treatment.

### 3.6 Assessment of inconsistency

The results of inconsistency assessments between direct and indirect estimates indicated that the overall level of each antithrombotic strategy satisfied the assumption of consistency (*p* >0.05). There were no significant differences among all comparisons (*p* >0.05). Supplementary Table S2 shows the details of loop-specific heterogeneity.

### 3.7 Meta-regression

The subgroup meta-regression analyses indicated that the device type did not substantially influence the occurrence of thrombosis and bleeding events (*p* >0.05 for each outcome). There was no significant difference regarding the outcomes of interest between patients who received the WATCHMAN device and patients who received the Amulet or ACP device (Supplementary Table S3).

### 3.8 Publication bias

Comparison-adjusted funnel plots were performed to test the publication bias among the enrolled studies. The results showed that the statistically symmetrical funnel plots did not indicate evidence of publication bias (Supplementary Figure S3). However, for the comparison of DAPT and VKA, only one study was included, which may make the assessment of publication bias somewhat unreliable.

## 4 Discussion

Transcatheter left atrial appendage occlusion has become an emerging, effective intervention for preventing stroke and embolic events in patients with NVAF (Reddy et al., 2013; Kirchhof et al., 2016; Boersma et al., 2019; Osmancik et al., 2020; Hindricks et al., 2021). However, the occurrence of DRT and stroke complications after the implantation of the left atrial occluder device has always been an unavoidable clinical problem for most doctors and device developers, causing great controversy (Reddy et al., 2013; Main et al., 2016; Boersma et al., 2019; Holmes et al., 2019). It is reported that the prevention of thrombosis may be an important segment of reducing the incidence of complications after LAAO (Tung et al., 2017; Dukkipati et al., 2018; Fauchier et al., 2018). However, the optimal antithrombotic strategies have not been adequately studied, which has aroused great concerns and heated discussions (Nakajima, 2022).

The present network meta-analysis, based on 10 observational studies involving 1,674 patients, observed no significant differences in the interested outcomes among all antithrombotic treatments. Furthermore, the SUCRA of our analysis indicated that DAPT was ranked the worst among all antithrombotic strategies due to the higher risk of stroke, DRT, and major bleeding events, while VKAs were ranked the preferred antithrombotic strategy; in addition, the efficacy and safety of DOACs were appreciable for LAAO patients.

Although the results of a few studies illustrated the safety and efficacy of administering DAPT after LAAO, they remain inconsistent (Chun et al., 2013). The results of a subgroup analysis of five studies by Søndergaard et al. (2019) also showed that patients who received APT treatments reported more DRT events compared with those who received DOAC treatments after LAAO. Moreover, the use of VKA has been limited due to its high requirements for patient compliance, narrow therapeutic window, and interaction with multiple foods and drugs (Shendre et al., 2018). Furthermore, previous studies deduced that DOACs play an important role in the treatment of patients who underwent LAAO (Asmarats et al., 2020; Li et al., 2020). Several clinical trials have demonstrated the efficacy and safety of DOACs in preventing post-PCI and stent thrombosis in NVAF patients and AF patients with coronary heart disease, particularly showing a lower incidence of major bleeding events than warfarin (VKA) (Cimmino et al., 2020). Therefore, DOACs are increasingly being used in antithrombotic strategies after LAAO. A multicenter, randomized, controlled trial comparing the efficacy and safety of apixaban (DOACs) and DAPT post-LAAO was conducted, which looked forward to adding evidence for the safety and efficacy of receiving DOACs or DAPT after LAAO (Flores-Umanzor et al., 2020).

Two landmark trials of LAAO, the PREVAIL trial and the PROTECT-AF trial, were published in 2014 and 2016, respectively, and mainly explored the efficacy and safety of using warfarin (VKA) as antithrombotic therapy in LAAO patients (Holmes et al., 2014; Main et al., 2016). These two large multicenter, randomized trials indicated that VKA followed by DAPT, was feasible for use in patients without anticoagulant contraindications post-LAAO. However, both the PROTECT-AF and PREVAIL trials did not enroll patients with contraindications and had controversial conclusions. Meanwhile, there is a lack of high-quality meta-analyses that explored different antithrombotic regimens. Meta-analyses, which have been published previously, included a total of 32 studies with 4,474 patients, indicating that DOACs have good prospects for development and may serve as alternatives to VKAs in the future. However, the study had several limitations that should not be overlooked; most of the studies included in the meta-analysis were single-arm studies and the level of evidence was not high; in addition, heterogeneity was analyzed, but no source of heterogeneity was identified for all-cause mortality (Li et al., 2020). Therefore, more extensive RCTs are needed to confirm the efficacy and safety of DOACs in post-LAAO patients. In summary, whether using VKAs or DOACs, the optimal antithrombotic strategy after LAAO requires extensive exploration.

In addition, to explore whether the device type plays an important role in postoperative outcomes during follow-ups, which remains controversial, we conducted a meta-regression in direct comparative subgroups using the proportion of the device type. The results of the subgroup meta-regression showed that the device type did not substantially influence the occurrence of thrombosis and bleeding events. Meanwhile, a real-world study compared the WATCHMAN and Amulet devices in an independent registry and concluded that the two devices showed similar efficacy and safety during long-term follow-ups (Saad et al., 2021).

Nevertheless, several limitations should be considered in this analysis. First, most of the studies enrolled in this network meta-analysis are observational studies. The lack of randomization in observational studies and poor transitivity among studies may lead to bias in the results of the network meta-analysis. Furthermore, most of the studies included reported event rates only based on the follow-up period, and we could not show the relationship between the events and time. In addition, there was a considerable gap in regard to the number of studies that included each antithrombotic regimen, and only one adapted study compared VKA and DAPT. Finally, this analysis just evaluated the efficacy and safety of different antithrombotic regimens, and the effect of individual drugs on postoperative outcome events was not considered.

## 5 Conclusion

Overall, no significant difference was found in the network meta-analysis among different antithrombotic strategies. Furthermore, the SUCRA indicated that DAPT is the worst antithrombotic strategy, while VKAs were the best. However, DOACs are a strategy worth considering due to their advantages of fixed-dose and no need for regular monitoring. This finding must be validated in larger prospective clinical studies.
