# Supplementary material for: Short-term antithrombotic strategies after left atrial appendage occlusion: a systematic review and network meta-analysis
Source: Front Pharmacol. 2023 Sep 1;14:1159857. doi: 10.3389/fphar.2023.1159857 (PMC10502722; doi:10.3389/fphar.2023.1159857)
Supplement: Supplementary file 1 [file DataSheet1.docx]

Supplementary Material

**Short-term antithrombotic strategies after left atrial appendage occlusion: A Systematic Review and Network Meta-Analysis**

Li-Man WANG^1,2†^, Yan CHEN^1,2†^, Li-Li XU^1,2^, Meng-Fei DAI^1,2^, Yi-Jun KE^3^, Bao-Yan WANG^1^, Lin ZHOU^1^, Ji-Fan ZHANG^4^, Zhang-Qi WU^5^, Yu-Jie ZHOU^6*^, Zhi-Chun GU^7*^, Hang XU^1*^

*** Correspondence:**

Yu-Jie ZHOU ([yujiezhoum@163.com](mailto:yujiezhoum@163.com))

Zhi-Chun GU ([guzhichun213@163.com](mailto:guzhichun213@163.com))

Hang XU ([njglyyxh@126.com](mailto:njglyyxh@126.com))

**Supplement materials**

**Figure S1.** Network plot for the network meta-analysis of all outcomes.

**Figure S2.** Pairwise meta-analysis of all outcomes.

**Figure S3.** Funnel plot for all outcomes.

**Table S1.** Quality assessment of included studies.

**Table S2.** Loop-specific heterogeneity for all outcomes.

**Table S3.** Results of subgroup meta-regression


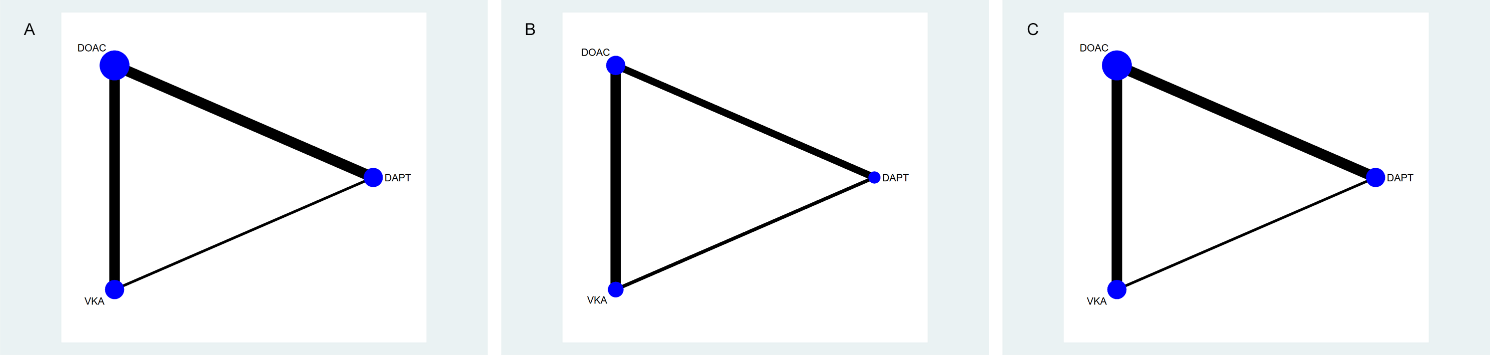


**Figure S1.** Network plot for the network meta-analysis of all outcomes. (A)strokes; (B) DRT; (C)major bleeding

**
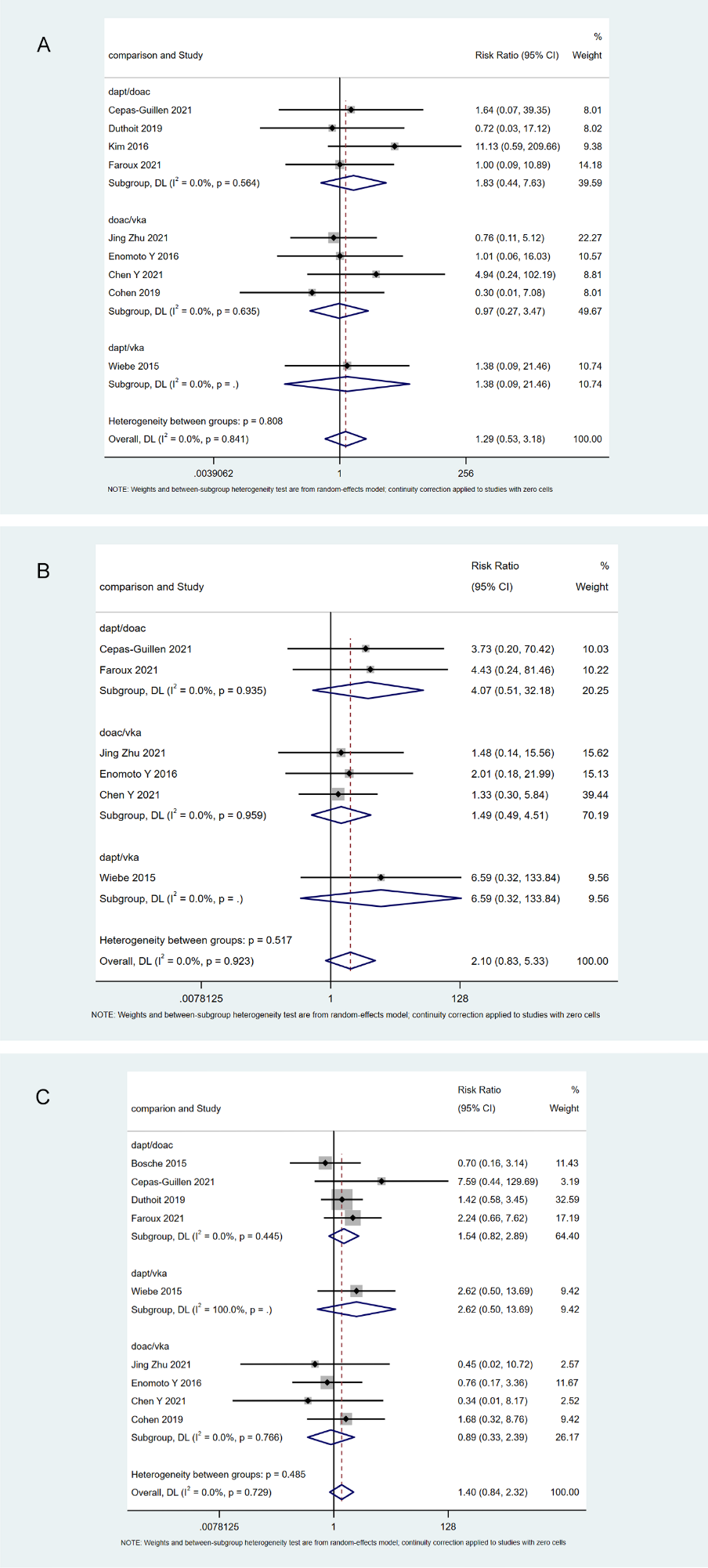
**

**Figure S2.** Pairwise meta-analysis of all outcomes. (A)strokes; (B) DRT; (C)major bleeding


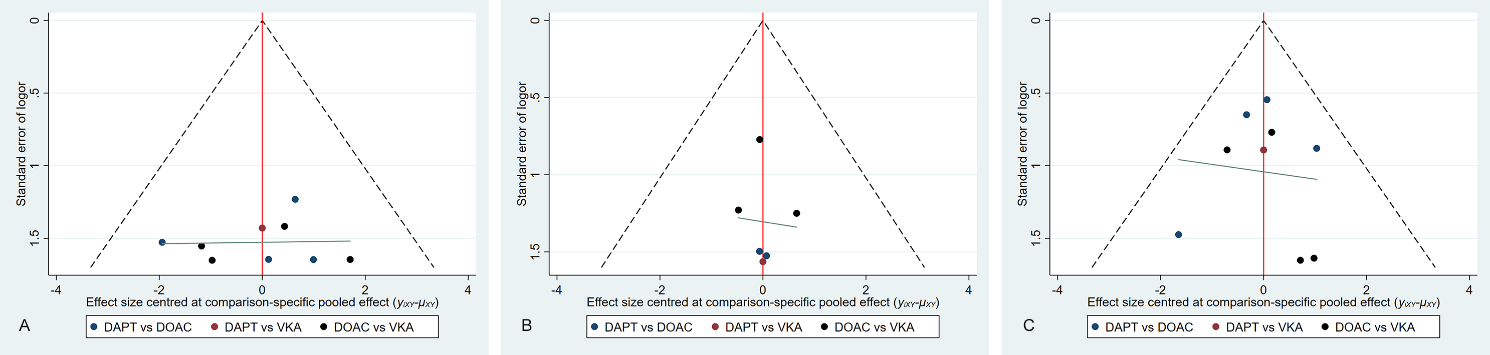


**Figure S3.** Funnel plot for all outcomes. (A)strokes; (B) DRT; (C)major bleeding.

**Table S1.** Quality assessment of included studies.

| **Quality assessment of comparative studies by the New-castle Ottawa Scale (NOS)** | | | | | | | | | |
| --- | --- | --- | --- | --- | --- | --- | --- | --- | --- |
| **Study** | **Selection** | | | | **Comparability** | **Outcome** |  |  | **Total score** |
|  | Representative- ness of the exposed cohort | Selection of the non-exposed cohort | Ascertainment  of exposure | Demonstration of outcome of interest was not present at start of study | On the basis of design or analysis | Assessment of outcome | Follow-up long enough for outcomes to occur | Adequacy of follow-up |  |
| Bosche 2015 | * | * | * | * | * | * | * | * | 8 |
| Cepas-Guillen 2021 | * | * | * | * | * | * | * | * | 8 |
| Duthoit 2019 | * | * | * | * | * | * | * | * | 8 |
| Faroux 2021 | * | * | * | * | ** | * | * | * | 9 |
| Kim 2016 | * | * | * | * |  |  | * | * | 6 |
| Wiebe 2015 | * | * | * | * | * | * | * | * | 8 |
| Chen Y 2021 | * | * | * | * | * | * |  | * | 7 |
| Cohen 2019 | * | * | * | * | ** | * | * | * | 9 |
| Enomoto Y 2016 | * | * | * | * | * | * | * | * | 8 |
| Jing Zhu 2021 | * | * | * | * | * | * |  | * | 7 |

**Table S2.** Loop-specific heterogeneity for all outcomes.

| Outcomes | Loop | IF | seIF | Z | P | 95%CI | τ^2^ |
| --- | --- | --- | --- | --- | --- | --- | --- |
| stroke | DAPT-DOACs-VKAs | 0.745 | 1.788 | 0.417 | 0.677 | (0.00, 4.25) | 0.000 |
| DRT | DAPT-DOACs-VKAs | 0.292 | 1.979 | 0.148 | 0.883 | (0.00, 4.17) | 0.000 |
| Major bleeding | DAPT-DOACs-VKAs | 0.655 | 1.095 | 0.598 | 0.550 | (0.00, 2.80) | 0.000 |

Abbreviations: DAPT, dual antiplatelet therapy; DOACs, direct oral anticoagulant; VKAs, vitamin K Antagonist.

**Table S3.** Results of subgroup meta-regression

| Outcomes | Comparison | Num. | _ES | Coef. | Std. Err. | t | P>\|t\| | 95% Conf. Interval | |
| --- | --- | --- | --- | --- | --- | --- | --- | --- | --- |
| Stroke | DAPT-DOAC | 4 | Watchman% | 3.206624 | 4.12659 | 0.78 | 0.518 | -14.54866 | 20.96191 |
|  | DOAC-VKA | 4 |  | -2.837363 | 2.496067 | -1.14 | 0.374 | -13.57707 | 7.902345 |
|  | VKA-DAPT | 1 |  | - | - | - | - | - | - |
| DRT | DAPT-DOAC | 2 | Watchman% | - | - | - | - | - | - |
|  | DOAC-VKA | 3 |  | - | - | - | - | - | - |
|  | VKA-DAPT | 1 |  | - | - | - | - | - | - |
| Major bleeding | DAPT-DOAC | 4 | Watchman% | -1.438056 | 1.299185 | -1.11 | 0.384 | -7.027999 | 4.151888 |
|  | DOAC-VKA | 4 |  | 1.608677 | 2.558366 | 0.63 | 0.594 | -9.399085 | 12.61644 |
|  | VKA-DAPT | 1 |  | - | - | - | - | - | - |
